# South African Dietitians' Knowledge and Perceptions of Food‐Drug Interactions and Factors Affecting It

**DOI:** 10.1111/jhn.70010

**Published:** 2025-01-13

**Authors:** Christie Megaw, Natascha Olivier, Werner Cordier

**Affiliations:** ^1^ Department of Pharmacology, School of Medicine, Faculty of Health Sciences University of Pretoria Pretoria Gauteng South Africa; ^2^ Department of Human Nutrition, School of Healthcare Sciences, Faculty of Health Sciences University of Pretoria Pretoria Gauteng South Africa

**Keywords:** dietitians, drug‐food interactions, food‐drug interactions, knowledge, perceptions, pharmacology

## Abstract

**Background:**

Dietitians ensure that patients receive tailored medical nutrition therapy to integrate with pharmacotherapy safely. Dietitians require a pharmacological understanding to prevent detrimental food‐drug interactions (FDIs). The study investigated dietitians' knowledge of FDIs and their information sourcing.

**Methods:**

A cross‐sectional online survey was conducted among registered South African dietitians to assess their knowledge of FDIs, the impact of food timing on drugs, and their sources of FDI information. The questionnaire included demographics, a 12‐question knowledge assessment, and a qualitative section on information sourcing. Data from 70 valid responses, collected between 2 August and 19 September 2022, were analysed statistically using analysis of variance and chi‐square tests to determine whether associations between knowledge scores and demographic factors were present.

**Results and Discussion:**

Out of 70 responses, most participants were female (97.1%) and 47.1% had over 10 years of experience. The participants primarily worked in the areas of dietetics related to chronic and lifestyle‐related disorders (75.7%) across various work settings, including in‐patient care (32.8%), out‐patient care (41.4%), and multi‐disciplinary team environments (31.4%). Although not generalisable due to the low response rate (70 out of the 304 required responses for a 5% margin of error), knowledge deficiencies were observed. A cumulative mean knowledge score of 38.3% was observed, with gaps identified for fundamental FDIs. Drug package inserts (55.7%) and clinical websites (68.6%) were primarily used to source information regarding FDIs; however, the former did not always provide sufficient information. Participants proposed that knowledge deficiencies could be overcome with further education, and the development and/or use of mobile applications or summarisations that elaborate on FDIs.

**Conclusion:**

Knowledge gaps and uncertainties were identified regarding fundamental FDIs; however, further research is needed to pinpoint the specific sources of these deficiencies and the factors influencing them. To improve dietitians' knowledge of FDIs and ensure alignment with their scope and standard of practice, undergraduate curricula should be bolstered and benchmarked to national needs to facilitate graduate development, and additional learning opportunities provided, such as webinars and continuing professional development (CPD), to allow for continuous education for practicing dietitians.

## Summary

- The study provides insights into potential knowledge deficiencies about food‐drug interactions in South African registered dietitians.
- Food‐drug interaction information sources, both academic and nonacademic, need to be supplemented with continuous professional development.
- Package inserts are often not available or feasible as a source of information on food‐drug interactions.

## 1 Introduction

A food‐drug interaction (FDI) is defined as a clinically significant effect arising from the interaction between a pharmaceutical agent and a specific food or food group [1]. The manifestation of these interactions varies based on the underlying pharmacokinetic and/or pharmacodynamic mechanisms [1]. There are several FDIs that have clinically significant repercussions for patient outcomes, which include altered therapeutic or adverse effect profiles, making them fundamental for healthcare practitioners to understand. Examples include the diminished anticoagulant properties of warfarin due to vitamin K‐rich foods (e.g., leafy greens) [2, 3], the chelation of tetracyclines by dairy products [2], digoxin's reduced absorption when consumed with fiber, and the hypertensive crisis triggered by monoamine oxidase inhibitors interacting with tyramine‐rich foods such as cheese and fermented products [2, 3]. As new medicines and food items enter the market, new interactions will invariably occur [1]. Understanding the potential interactions between food items and pharmacotherapy remains important to successful treatment in both patients and treating practitioners, such as dietitians.

FDIs are particularly relevant to dietitians' scope and standard of practice due to the practitioner's key role in nutritional guidance and therapy [1, 3]. As defined by Section 33 [1] of the Medical, Dental, and Supplementary Health Service Professions Act of South Africa, 1974 (Act No. 56 of 1974), dietitians ensure patients' safety and nourishment, provide tailored medical nutrition therapy upon referrals, and educate the community and individuals on healthy lifestyles and nutrition [4]. Dietitians' foundational training includes pharmacology integrated or separate from their core professional modules depending on the South African university [4]. However, little is known about dietitians' knowledge of the prevalence, risk, and management of FDIs. Only localised FDI knowledge studies was assessed for other countries, often in multiprofessional format, impacting the generalisability of their results: California (United States, 2006) [5]; Mysore (India, 2012) [6]; Palestine (Western Asia, 2016 to 2017) [7]; KwaZulu Natal (South Africa, 2018 to 2019) [2]; Riyadh (Saudi Arabia, 2021‐2022) [8]; the eastern region of Saudi Arabia (2022) [9]; and Harari Regional State (Eastern Ethiopia, 2022) [10]. Further education was recommended given the lack of FDI knowledge in several healthcare professions, including doctors [2, 6, 9, 10], nurses [2, 9, 10], pharmacists [2, 7, 10], and dietitians [2, 5, 6]. Although pharmacists [7] and dietitians [2] perceived their own FDI knowledge to be high, objective assessments of their FDI knowledge did not necessarily support their perceptions. Insufficient FDI knowledge was observed for dietitians within the KwaZulu Natal region of South Africa [2], though results could not be generalised to the national population given the sampling strategy and low sample size (14 participants). Given the scarcity of research focusing on FDI knowledge in healthcare professionals, it is unclear what is understood among dietitians, and where they source their information.

The study aimed to investigate the knowledge that South African dietitians have on selected clinically relevant FDIs, where information is generally sourced, and to identify ways in which their knowledge can be expanded.

## 2 Method

### 2.1 Study Design, Questionnaire Design and Validation

An online Qualtrics XM (Qualtrics LLC) questionnaire was used in a cross‐sectional, descriptive study design, allowing registered dietitians from across South Africa to conveniently complete the questionnaire anonymously and asynchronously between 2 August 2022 and 19 September 2022 after distribution by the Association for Dietetics in South Africa (ADSA) and social media. The questionnaire design was guided by published literature [2, 6, 11] and interactions cross‐checked with each other to reduce conflicting information and increase the accuracy of statements. The questionnaire consisted of four sections: A) demographics (background, education, working environments); B) knowledge of selected FDIs; C) knowledge of the timing of drugs relative to food; and D) perceptions of preparedness, sourcing of information, and expansion of knowledge (Supplementary File).

The demographic information collected in Section A included gender, age, registration status with the HPCSA, years of experience, undergraduate institution in dietetics (results blinded due to ethical concerns), method of undergraduate training in pharmacology, highest qualification, employment status, work settings (multiple selections possible), and primary areas of dietetics practice (multiple selections possible). Sections B and C consisted of multiple‐choice questions to determine participants' FDI knowledge using quantitative means. Questions comprised general knowledge of FDIs, susceptible populations and timing considerations, where those with multiple answers were assessed via negative marking (relative to the total score of the question) to account for incorrect selections. Questions concerning FDIs between medicines and food items featured a drug with five items of food that could be interacted with. Participants were able to select whether the foodstuff would or would not interact with the drug, or whether they were uncertain. Participants were scored per correct answer to aggregate their score between the sections. Section D comprised both multiple‐choice questions and open text options to obtain quantitative and qualitative data, where the latter was used to enrich and elaborate on findings. In line with the study Baig et al. 2020, a score of less than 50% is considered poor, 51%–75% is considered moderate, and 76%–100% is considered good [12]. The same approach was used when dealing with the levels of uncertainty.

The questionnaire was content‐validated by three pharmacologists and one dietitian, and face‐validated by six dietitians. During validation, editorial and content recommendations were adopted to ensure readability and clarity.

### 2.2 Sample Group

Eligible dietitians registered with the Health Professions Council of South Africa (HPCSA), and able to read and complete the English questionnaire were included in the study. A minimum of 304 registered South African dietitians was required based on the assumed total of registered dietitians in South Africa to allow for a 5% margin of error (calculated by Raosoft, http://www.raosoft.com/samplesize.html), however, as only 70 valid responses were obtained the margin of error increased to 11.23% and reduced the generalisation of results.

### 2.3 Questionnaire Distribution

A non‐probability purposive selection approach using members of the ADSA was employed. The ADSA newsletter was sent out via email containing the survey supported by distribution through the South African “Dietetics is a Profession” social media page. A biweekly reminder was sent out along with the following ADSA newsletter. A short, embedded video was included in the distributed email to promote the relevance of the study. Participants were required to give informed consent by selecting the appropriate option on the cover page of the online questionnaire, before being allowed to proceed with the questionnaire. The questionnaire took between 15 and 20 min for participants to complete.

### 2.4 Data Collection and Analysis

Data were collected using Qualtrics and exported as a Statistical Package for the Social Sciences (SPSS) file for descriptive and statistical analysis. Statistical analysis included one‐way analysis of variance (ANOVA) with a post‐hoc Tukey's test for means comparisons, and chi‐square analysis to determine association between knowledge and area of practice. A *p*‐value < 0.05 was used to indicate significance. Internal consistency was calculated for Sections B and C, which achieved an acceptable Cronbach‐α value of 0.886.

Qualitative data were analysed inductively using Excel through a three‐step process: data were first read for understanding; thereafter coded; and lastly allocated to themes. Coding was facilitated by a code book that was established between the research team.

### 2.5 Ethical Consideration

Approval was provided by the Faculty of Health Sciences Research Ethics Committee of the University of Pretoria (REC 202/2022). Permission was sought and obtained from ADSA to make use of their distribution platforms, including their weekly newsletter and Facebook group. Informed consent was obtained from participants via the cover page of the online questionnaire. To ensure participant anonymity in accordance with the 2013 Protection of Personal Information Act, participants were made aware that all information gathered throughout the study would be anonymous, with no way to link responses to a specific person. Institutional names were blinded during reporting to ensure anonymity.

## 3 Results

### 3.1 Demographics

Of the 70 participants (Table 1), the majority were female (97.1%) and over 30 years old (62.9%). The most frequently selected range for years' experience was 5 to 9 years of experience (27.1%), with 47.1% presenting with more than 10 years of experience. The participants' primary work setting related to chronic and lifestyle‐related disorders (75.7%), with 52.9% and 41.4% working in private and out‐patient care. Most participants received some form of pharmacological training (85.7%) with an approximate equal distribution between integrated or stand‐alone modules.

**Table 1:** The association between demographic characteristics and cumulative knowledge of participants (n = 70) as measured by means‐comparison (gender, age, pharmacology training, and experience) and chi‐square analysis (work setting and area). The chi‐square analysis was done per area of work, so it compares the involvement in the area versus not being involved in it. Significance was defined as a *p*‐value < 0.05.

Demographics		Frequency (Percentage)	Mean score ± standard deviation (out of 48)	*p*‐value
Gender	Male	2 (2.9)	24.8 ± 13.9	0.859
	Female	68 (97.1)	18.2 ± 7.4	
	Nonbinary	0 (0.0)	n.a	
	Prefer not to say	0 (0.0)	n.a	
Age (years)	21 – 25	10 (14.3)	19.2 ± 7.1	0.499
	26 – 30	16 (22.9)	16.1 ± 5.4	
	31 – 35	13 (18.6)	17.3 ± 10.1	
	36 – 40	10 (14.3)	18.0 ± 9.0	
	40+	21 (30.0)	23.5 ± 5.4	
Highest level of education	Bachelors	40 (57.1)	17.5 ± 8.4	0.173
	Masters/Doctorate**	20 (28.6)	18.6 ± 6.5	
Work setting*	In‐patients	23 (32.8)	18.5 ± 7.5	
	Outpatients	29 (41.4)	18.6 ± 6.9	0.630
	Multi‐disciplinary	22 (31.4)	19.8 ± 6.8	0.560
	Tertiary hospital	11 (15.7)	20.5 ± 5.6	0.425
	Small rural hospital	7 (10.0)	19.8 ± 8.6	0.158
	Public sector	20 (28.6)	17.5 ± 7.5	0.363
	Private sector	37 (52.9)	17.6 ± 7.9	0.410
	Chronic/lifestyle diseases	53 (75.7)	18.5 ± 7.8	0.354
Areas of dietetics predominantly worked in*	Critical care	20 (28.6)	19.4 ± 8.9	0.522
	Paediatrics	29 (41.4)	18.1 ± 7.5	0.519
	Renal nutrition	10 (14.3)	22.5 ± 9.1	0.441
	Oncology	13 (18.6)	17.6 ± 7.5	0.274
	Infectious diseases	8 (11.4)	18.9 ± 8.5	0.265
	Lactation	17 (24.3)	18.3 ± 8.9	0.070
	Neonatology	5 (7.1)	17.9 ± 9.0	0.385
	Separate pharmacology module	31 (44.3)	18.7 ± 6.4	0.308
Pharmacology training	Integrated pharmacology module	29 (41.4)	19.0 ± 8.7	0.319
	No pharmacology training	2 (2.9)	19.7 ± 2.8	
	Unsure/cannot recall	8 (11.4)	14.3 ± 7.9	
	0 – 4	18 (25.7)	19.4 ± 7.9	
Experience as dietitian (years)	5 – 9	19 (27.1)	17.7 ± 6.3	0.193
	10 – 14	11 (15.7)	16.3 ± 6.7	
	15 – 19	13 (18.6)	12.4 ± 5.3	
	20+	9 (12.9)	22.5 ± 7.8	
Institution of training	University 1	8 (11.4)	15.7 ± 7.3	0.352
	University 2	4 (5.7)	22.4 ± 10.4	
	University 3	18 (25.7)	16.8 ± 7.9	
	University 4	5 (7.1)	20.1 ± 6.8	
	University 5	10 (14.3)	22.9 ± 8.4	
	University 6	3 (4.3)	20.8 ± 12.2	
	University 7	17 (24.3)	17.3 ± 5.0	
	University 8	2 (2.9)	19.5 ± 2.1	

* choose all that apply question,

** only one dietitian with a doctorate responded, n.a. not applicable.

### 3.2 Knowledge of Fdis

The overall mean cumulative score (out of 48) was 18.3 ± 7.6 (38.1%), and 57.1% of the participants scored below the mean. The mean score was therefore considered poor and as more than half the participants scored below the mean the overall knowledge of the dietitians on FDIs can be considered poor [13]. No significant associations were observed between the cumulative mean score of each demographic stratification (Table 1). The questions were grouped into two sections: Section B (selected FDIs; section total = 42; maximum score achieved = 29.67; minimum score achieved = 6; mean score ± SD = 17.6 ± 6.9) and Section C (timing of food‐ intake relative to drugs; section total = 6; maximum score achieved = 4.5; minimum score achieved = 0; mean score ± SD = 0.7 ± 1.1).

Section B covered three themes: general questions (pharmacokinetics, pharmacodynamics, nutritional status); age groups most affected by FDIs and food‐drug specific questions (angiotensin‐converting enzyme inhibitors [ACE‐inhibitors], alcohol, digoxin, grapefruit, monoamine oxidase [MAO]‐inhibitors, tetracyclines, warfarin). Participants scored well in the general questions related to the clinical significance and general pharmacokinetic and pharmacodynamic mechanisms (mean score ± SD = 5.4 ± 0.8). Knowledge deficiencies were observed for age groups most affected by FDIs and specific FDI questions (Table 2). Few participants obtained full marks for each individual drug: warfarin (*n* = 3, 4.3%), angiotensin‐converting enzyme inhibitors (*n* = 9, 12.9%), monoamine oxidase inhibitors (*n* = 3, 4.3%), and alcohol (*n* = 2, 2.9%).

**Table 2:** The minimum score, maximum score, and mean score obtained by the participants for each question.

Questions	Statistics		
	Minimum score achieved	Maximum score achieved	Mean score ± standard deviation
General questions of food‐drug interactions (possible score = 6)	3.0	6.0	5.4 ± 0.8
Most affected age groups (possible score = 1)	0.0	1.0	0.4 ± 0.4
Warfarin (possible score = 5)	0.0	5.0	2.5 ± 1.2
Tetracyclines (possible score = 5)	0.0	4.0	0.9 ± 1.1
ACE‐inhibitors (possible score = 5)	0.0	5.0	2.3 ± 1.8
MAO‐inhibitors (possible score = 5)	0.0	5.0	1.7 ± 1.8
Digoxin (possible score = 5)	0.0	3.0	0.9 ± 1.2
Grapefruit (possible score = 5)	0.0	4.0	1.8 ± 1.9
Alcohol (possible score = 5)	0.0	5.0	1.7 ± 1.4
Section B Total	6.0	29.67	17.6 ± 6
Timing of drugs relative to food and nutrient considerations (possible score = 3)	0.0	1.8	0.2 ± 0.4
Timing of drugs relative to food (possible score = 3)	0.0	3.0	0.5 ± 0.8
Section C Total	4.5	0.0	0.7 ± 1.1
Total overall	34.5	6.0	18.3 ± 7.6

Individual scores per question are presented in Table 3. High levels of uncertainty of the interaction were seen between tetracyclines and dairy products (51.4%), tetracyclines and iron‐containing foods (nuts [68.6%] and red meat [64.3%]), ACE‐inhibitors and bananas (21.4%), and MAO‐inhibitors and fermented products (cheese [51.4%], sourdough bread [58.6%] and processed meats [52.9%]). Alcohol in general should be avoided with any drugs, however, there was a high level of uncertainty on whether it should be co‐administered with select drugs.

**Table 3:** The participant selections for specific FDI questions, which allowed for the selection of yes, uncertain, or no. Bolded values indicate the most frequently selected options.

Drug/food/drink	Interacts with… (correct answer)	Correct *n* (%)	Incorrect *n* (%)	Uncertain *n* (%)
Warfarin	Garlic (yes)	16 (22.9)	**36 (51.4)**	18 (25.7)
	Cranberry (yes)	20 (28.6)	**32 (45.7)**	18 (25.7)
	Leafy greens (yes)	**64 (91.4)**	2 (2.9)	4 (5.7)
	Beets (no)	**34 (48.6)**	10 (14.3)	26 (37.1)
	Strawberry (no)	**47 (67.1)**	0 (0.0)	23 (32.9)
Tetracyclines	Beans (yes)	2 (2.9)	19 (27.1)	**49 (70.0)**
	Dairy products (yes)	30 (42.9)	4 (5.7)	**36 (51.4)**
	Nuts (yes)	2 (2.9)	20 (28.6)	**48 (68.6)**
	Red meat (yes)	4 (5.7)	21 (30.0)	**45 (64.3)**
	Chicken (no)	24 (34.3)	1 (1.4)	**45 (64.3)**
ACE‐inhibitors	Oranges (yes)	23 (32.9)	18 (25.7)	**29 (41.4)**
	Bananas (yes)	**48 (68.6)**	7 (10.0)	15 (21.4)
	Peaches (no)	26 (37.1)	7 (10.0)	**37 (52.9)**
	Pears (no)	29 (41.4)	3 (4.3)	**38 (54.3)**
	Apples (no)	33 (23.6)	2 (2.9)	**35 (50.0)**
Digoxin	Avocados (yes)	4 (5.7)	20 (28.6)	**46 (65.7)**
	Whole‐grain bread (yes)	13 (18.6)	15 (21.4)	**42 (60.0)**
	Celery (yes)	5 (7.1)	14 (20.0)	**51 (72.9)**
	Watermelon (no)	17 (24.3)	4 (5.7)	**49 (70.0)**
	White rice (no)	25 (35.7)	1 (1.4)	**44 (62.9)**
Grapefruit	Simvastatin (yes)	**44 (62.9)**	6 (8.6)	20 (28.6)
	Nifedipine (yes)	26 (37.1)	12 (17.1)	**32 (45.7)**
	Paracetamol (yes)	9 (12.9)	28 (40.0)	**33 (47.1)**
	Fexofenadine (yes)	10 (14.3)	26 (37.1)	**34 (48.6)**
	Zanamivir (no)	10 (14.3)	19 (27.1)	**41 (58.7)**
MAO‐inhibitors	Matured cheese (yes)	29 (41.4)	5 (7.1)	**36 (51.4)**
	Sourdough bread (yes)	13 (18.6)	16 (22.9)	**41 (58.6)**
	Processed meats (yes)	28 (40.0)	5 (7.1)	**37 (52.9)**
	Cottage cheese (no)	20 (28.6)	9 (12.9)	**41 (58.6)**
	Pasta (no)	28 (40.0)	1 (1.4)	**41 (58.6)**
Alcohol	Antiretrovirals (yes)	**34 (48.6)**	5 (7.1)	31 (44.3)
	Antihistamines (yes)	**32 (45.7)**	8 (11.4)	30 (42.9)
	Warfarin (yes)	24 (34.3)	11 (15.7)	**35 (50.0)**
	Aspirin (yes)	30 (42.9)	7 (10.0)	**33 (47.1)**
	Paracetamol (yes)	23 (32.9)	19 (27.1)	**28 (40.0)**

There were significant associations between certain areas in which dietitians work and knowledge components (Table 4), for example, where higher mean scores were achieved by individuals working within chronic/lifestyle disorders for monoamine oxidase inhibitor interactions (mean score ± SD = 1.8 ± 1.8, *p*‐value = 0.017) when compared to participants working in other areas of dietetics. Conversely, lower mean scores were achieved by those working in paediatrics for interactions between food items and warfarin (mean score ± SD = 2.2 ± 0.8, *p*‐value = 0.014) when compared to participants working in other areas of dietetics.

**Table 4:** The association between knowledge components and area(s) of dietetics predominantly worked in. Significance was defined as a *p*‐value < 0.05 (*), where comparison was made between the knowledge component and all areas of dietetics predominantly worked in, but only significant comparison have been included in the table. Bolding applies to the highest score in the group.

Area	Whether participants were involved with the area	Frequency (percentage)	Knowledge Component	Mean score ± standard deviation	*p*‐value
Chronic/lifestyle disorders	Yes	53 (75.7)	Monoamine oxidase inhibitors	**1.8** ± **1.8**	0.017*
	No	17 (24.3)		1.4 ± 1.5	
Critical care	Yes	20 (28.6)	Timing of drugs	**0.7** ± **0.9**	0.021*
	No	50 (71.4)		0.4 ± 0.7	
	Yes	8 (11.4)	Timing and nutrient considerations of drugs	0.2 ± 0.4	0.002*
	No	62 (88.6)		**0.4** ± **0.6**	
	Yes	8 (11.4)	Section C total: (Knowledge of timing of Food Intake Relative to Drugs)	**1.1** ± **1.4**	0.009*
	No	62 (88.6)		0.7 ± 1.0	
	Yes	10 (16.7)	Monoamine oxidase inhibitors	1.5 ± 1.6	0.023*
	No	60 (83.3)		**2.9** ± **2.1**	
	Yes	10 (16.7)	Alcohol and drug interactions	**2.5** ± **1.2**	0.005*
	No	60 (83.3)		1.5 ± 1.8	
	Yes	10 (16.7)	Timing of drugs	**1.2** ± **1.2**	0.002*
	No	60 (83.3)		0.4 ± 0.6	
Lactation	Yes	17 (24.3)	Tetracyclines	**1.2** ± **1.4**	0.034*
	No	53 (75.7)		0.8 ± 0.9	
Paediatrics	Yes	29 (41.4)	Warfarin	2.2 ± 0.8	0.014*
	No	41 (58.6)		**2.8** ± **1.4**	
Oncology	Yes	13 (18.6)	Digoxin	**1.0** ± **0.9**	0.038*
	No	57 (81.4)		0.9 ± 1.2	

### 3.3 Perceived Frequency of Clinically‐Significant Interactions, Information Sources, and Knowledge Expansion

Participants believed that they seldom (30%) or never (32.9%) encountered clinically significant FDIs in practice (Table 5). The main information sources for FDIs were drug package inserts (55.7%) and clinical websites (68.6%) (Table 5). Only 30% of participants used drug package inserts during consultations, but when package inserts were consulted, 52.9% indicated that it did not provide sufficient information about interactions. Participants alluded to package inserts‘ insufficiency: [P5] ‘*Very few mention specific food drug interactions, if any*.’ or [P33] ‘*I believe there is a lot of extensive information on, but not really a lot about the food‐drug interactions*’. Although many participants (42.9%) always reviewed their patients' medication lists, only 42.9% occasionally requested that their patients bring their medications to the consultations.

**Table 5:** Participant feedback regarding the frequency of clinically significant FDIs, where information is sourced, whether dietitians look at patients’ medication and whether dietitians use package inserts during consultations. Bolding indicates the most frequent response.

Question		Possible options	Frequency (percentage)
Frequency of clinically significant FDI encounters		**Never**	**23 (32.9)**
		Infrequently (once or twice a year)	21 (30.0)
		Frequently (monthly)	9 (12.9)
		Almost always (weekly)	5 (7.1)
		Always (daily)	2 (2.9)
Source of information for FDIs		Drug package inserts	39 (55.7)
		Textbooks	24 (34.3)
		**Clinical websites**	**48 (68.6)**
		Social media	1 (1.4)
		Mobile applications	8 (11.4)
Regarding the use of package inserts in consultations	Review of medication list of patients during consultations	**Always**	**30 (42.9)**
		Sometimes	17 (24.3)
		Never	2 (2.9)
		Not applicable	3 (4.3)
	Patients required to bring medication to consultations	Always	7 (10.0)
		**Sometimes**	**30 (42.9)**
		Never	13 (18.6)
		Not applicable	9 (12.9)
	Package inserts are used during consultations	Always	2 (2.9)
		Sometimes	19 (27.1)
		**Never**	**30 (42.9)**
		Not applicable	7 (10.0)
	Package inserts perceived to provide sufficient information	Always	6 (8.6)
		**Sometimes**	**37 (52.9)**
		Never	6 (8.6)
		Not applicable	10 (14.3)

### 3.4 Qualitative Data on Information Sources and Knowledge Expansion

Participants who encountered a greater frequency of FDIs were involved with treating patients with chronic diseases (such as diabetes mellitus and hypertension), or with oncopharmaceutics. Participants who did not encounter interactions frequently postulated that it was related to patients being well‐educated, having relatively uncomplicated disease profiles, or being unable to diagnose or recognise interactions (Table 6; Theme: Frequency of FDI encounters affected by patient, disease, and medication type).

**Table 6:** Qualitative data on information sources and knowledge expansion of FDIs.

Theme	Subtheme	Exemplar quotation(s)
Frequency of FDI encounters affected by patient, disease, and medication type	Chronic conditions and pharmacotherapy yield more FDIs	[P7] ‘I work in a clinic at least once a week. Most of which are antihypertensive drugs, ARVs [*antiretrovirals*], diabetic medication, TB [*tuberculosis*] medication’
		[P28] ‘Almost always as most medications are affected by the food a patient consumes as well as if they take the medication with or without meals’
		[P57] ‘Most patients are aware of the interactions or are not taking a lot of different medication.’
		[P89] ‘I work with outpatient [*sic*] who generally have no chronic illness or are not taking many chronic meds’
	Difficulty in diagnosing or recognising FDIs	[P34] ‘I think mostly due to ignorance. If my knowledge was better I might find it more often’
Variable levels of pharmacological education and information sourcing	Academic education available, but little training thereafter	[P11] ‘We had pharmacology (2 modules) in 3rd year and also had to elaborate on this in our practical medical nutrition cases. No further training after university.’
	Information sourced from peers or academic material	[P46] ‘pharmacist’ or [P89] ‘colleagues'
		[P11] ‘Old “cheatsheets” from university’
		[P82] ‘Drug interactions manual provided from the university I studied at’
Variable use of package inserts during consultations	Package inserts and medication lists used to inform the practitioner	[P24] ‘I ask the patient to provide me a list of their Rx [*treatment*] prior the consultation, to give me time to do research on their medication’
		[P5] ‘If the patient mentions medication that is causing problems, or wanting to know which medication they are using for DM [*diabetes mellitus*], HPT [*hypertension*], etc.’
		[P44] ‘If the script is unavailable or the patient doesn't know their medication. I often rebook them on the day they collect medication to make sure that they have the right meds.’
	Package inserts not always useful due to unavailability or lack of relevant information	[P35] ‘Patients rarely bring the pill box with the package insert still inside with to a consultation‐ they usually throw the insert away’
		[P5] ‘Very few mention specific food drug interactions, if any.’
		[P33] ‘I believe there is a lot of extensive information on, but not really a lot about the food‐drug interactions’
Further education on FDIs required	Participants feel ill‐equipped to manage FDIs at times	[P31] ‘I don't feel I have enough knowledge of each relevant drug‐nutrient interaction that I may expect to see’
		[P7] ‘I don't know where to find the correct and reliable information. What resources are available
	Training or reference material may support education	[P33] ‘Pharmaceutical companies can host CPD [*continuing professional development*] events and webinars…’
		[P5] ‘Easily accessible information, such as a website/app, to access for all available drugs. This makes it easier to find reliable information on the go, during a patient consult for example…’

Although some participants indicated that they had received training on FDIs as part of their undergraduate programme, further FDI education was needed and often required supplementation with academic resources (Table 6; Theme: Variable levels of pharmacological education and information sourcing). Apart from the sources mentioned in Table 5, other healthcare professionals were also consulted on FDIs. Participants that looked at their patients' medication list indicated that they did so to determine which medications their patients were on or only when concerned about particular medications. The main reasons for requesting medication lists or package inserts to be provided were due to patients being unable to recall their medications, not having the medication with them, or participants requiring time to prepare for their consultations. Participants did not use drug package inserts mainly as they were unavailable or lacked pertinent information on FDIs (Table 6; Theme: Variable use of package inserts during consultations).

Participants that felt equipped to deal with FDIs, only felt comfortable doing so when resources were consulted. Participants that were less comfortable did not feel they had sufficient knowledge of either the FDIs themselves or on where to source information. Participants indicated that knowledge could be improved through bolstered or additional training, for example, continuing professional development, webinars, reference materials, or interaction manuals/applications (Table 6; Theme: Further education on FDIs required).

## 4 Discussion

The knowledge of dietitians of several fundamental, clinically significant FDIs were determined in this study, for example vitamin K‐rich foods and warfarin (diminished anticoagulant properties), tetracyclines' chelation with dairy products (reduced therapeutic effect), digoxin's interaction with fibre (reduced therapeutic effect) and monoamine oxidase inhibitors and tyramine‐rich foods (increase adverse effects). In line with similar studies conducted on healthcare professionals [2, 5, 6, 7, 8, 9, 10, 11], the participants did not score well on the drug‐specific question and noted uncertainty of whether interactions may be present. This suggests that dietitians' confidence in their knowledge of such interactions were low and that supplementary education would be required. For instance, while the knowledge of interactions between warfarin and leafy greens and between ACE inhibitors and bananas was high, there was considerable uncertainty about other potential interactions within these categories. Furthermore, only a small number of dietitians could identify interactions involving digoxin and tetracycline, and few correctly identified interactions between ACE inhibitors, grapefruit, and alcohol. Dietitians also referred to a few other notable interactions in their qualitative feedback, which may suggest that they deal with them more specifically in their area of work (such as medication for diabetes, hypertension, tuberculosis, and thyroid diseases). In line with Osuala et al. [2] dietitians in the current study scored well for general questions related to the clinical significance and general pharmacokinetic and pharmacodynamic mechanisms. In line with other studies, including other healthcare professionals, there was a high level of uncertainty or inability to identify the age groups most affected by FDIs, which include pre‐term babies, paediatrics, and geriatrics due to altered physiological processes affecting pharmacokinetic properties [9, 13, 14]. In contrast, Syed Snr et al. had a low level of uncertainty when looking at most affected age groups [8].

No association between the demographic factors of participants and their knowledge was seen. Based on reports by Osuala et al. [2] Degefu, et al. [10] Alhubail, et al. [9] Radwan et al. [7] and Benni et al. [6] it's unclear how work experience impacts knowledge. How pharmacology was presented and at which institution had no statistically significant effect on knowledge.

Some participants indicated that they did make use of patient information leaflets, however, although the information to be included in these leaflets is regulated, the information was very often insufficient or lacking when used by these participants. This is in line with what other studies found: although there are laws and regulations in place to regulate the information and ensure that it is included, it was very often still insufficient or lacking [15, 16].

Knowledge deficiencies for selected FDIs were identified, which is consistent with findings from similar studies mentioned previously [2, 5, 6, 7, 8, 9, 10, 11]. As dietitians felt that they were not equipped to manage FDIs due to limited training or exposure to it within their clinical practice, further research is needed to determine the underlying factors that decrease their knowledge and/or confidence in managing such interactions. Dietitians further proposed several platforms to promote FDI knowledge development, such as additional training or a reference resource. Given the importance of managing interactions that may occur with pharmacotherapy, as per their scope of practice, strengthening of knowledge in this domain should be done to ensure competency attainment.

Given the small sample size, generalisation was impacted, but it does infer a knowledge deficiency and areas of improvement that can be targeted for the assessed population. As stratification further decreased the sample size within cohorts, it reduced the statistical power of the comparisons. Although common FDIs were selected, some may be outside of dietitians' direct practice, and thus be drugs that are not frequently encountered in their working environment, falling outside of their level of expertise. Some food items that are likely to be used in lower quantities, such as garlic, may also have been construed by participants as supplements with higher doses, thus altering the perception of the potential interaction. Future studies will need to specify such aspects more clearly to participants. The questions were also limited to determining the interaction, but did not assess the intensity of the interaction or the form in which the reaction presents, which may have biased participants' opinions on its significance or the presence thereof. Participants were not provided with the correct answers to allow for professional development, and thus a memorandum or related FDI information source should be included in future research endeavours.

## Author Contributions

**Christie Megaw:** conceptualization (equal), data curation (lead), formal analysis (lead), investigation, methodology (equal), project administration (lead), resources (lead), visualization (lead), writing‐original draft preparation. **Natascha Olivier:** conceptualization (equal), methodology (supporting), resources (supporting), supervision (supporting), validation (supporting), writing‐review and editing (equal). **Werner Cordier:** conceptualization (equal), data curation (supporting), formal analysis (supporting), methodology (equal), project administration (supporting), resources (supporting), supervision (lead), validation (lead), visualization (supporting), writing‐review and editing (equal).

## Ethics Statement

The study was approved by the Faculty of Health Sciences Research Ethics Committee of the University of Pretoria (REC 202/2022). The study was performed in accordance with the ethical standards as laid down in the 1964 Declaration of Helsinki and its later amendments or comparable ethical standards. Informed consent was obtained from all participants for being included in the study.

## Conflicts of Interest

Christie Megaw, Natascha Olivier and Werner Cordier declare no conflict of interest.

### 1 Peer Review

The peer review history for this article is available at https://www.webofscience.com/api/gateway/wos/peer-review/10.1111/jhn.70010.

## Supporting information

Supporting information.

## Data Availability

The data that support the findings of this study are available from the corresponding author upon reasonable request.
