# Supplementary material for: South African Dietitians' Knowledge and Perceptions of Food‐Drug Interactions and Factors Affecting It
Source: J Hum Nutr Diet. 2025 Jan 13;38(1):e70010. doi: 10.1111/jhn.70010 (PMC11729995; doi:10.1111/jhn.70010)
Supplement: Supplementary file 1 — Supporting information. [file JHN-38-0-s001.docx]

**Questionnaire administered to participants post-consent**

1. What is your gender?

- Male
- Female
- Non-binary/third gender
- Prefer not to say

1. What is your age (in years)?

- 21-25
- 26-30
- 31-35
- 36-40
- 40+

1. Are you registered with the Health Professions Council of South Africa as a dietitian?

- Yes
- No

1. How many years of experience (in years) as a dietitian do you have?

- 0-4
- 5-9
- 10-14
- 15-19
- 20+

1. At which institution did you obtain your undergraduate degree in dietetics?

- [Institutions and order blinded]
- An institution in South Africa not listed (please specify)
- An institution outside South Africa (please specify)

1. How was pharmacology incorporated into your undergraduate training?

- Separate pharmacology module
- Pharmacology integrated in another module (such as therapeutic nutrition)
- No pharmacology training provided
- Unsure/cannot recall
- Other (please specify)

1. What is your highest qualification?

- Bachelors
- Masters
- Doctorate
- Other (please specify)

1. Are you employed full-time or part-time as a dietitian?

- Full-time
- Part-time

1. In which setting(s) do you work? (multiple answers can be selected)

- In-patients
- Out-patients
- As part of a multi-disciplinary team
- In a tertiary hospital
- Small rural hospital
- Public sector
- Private sector
- Other (please specify)

1. Which area(s) of dietetics do you predominantly work in or identify with? (multiple answers can be selected)

- Chronic/lifestyle diseases
- Critical care
- Paediatrics
- Renal nutrition
- Oncology
- Infectious diseases
- Lactation
- Neonatology
- Other (please specify)

1. Please select the most correct answer, to the best of your knowledge, without consulting additional resources. In other words, select the answer that is based on existing knowledge.

| **Question** | **Yes** | **Not sure** | **No** |
| --- | --- | --- | --- |
| Food-drug interactions can lead to clinically-significant outcomes |  |  |  |
| Drink-food interactions can lead to clinically-significant outcomes |  |  |  |
| Food can affect the pharmacokinetics (the processes by which drugs are absorbed, distributed in the body, localised in the tissues, and excreted) of drugs |  |  |  |
| Food can affect the pharmacodynamics (the mechanisms by which drugs influence the physiological system, including dose-responses) of drugs |  |  |  |
| The nutritional status of a patient can be affected by certain drugs |  |  |  |
| The nutritional status of the patient can affect how drugs work |  |  |  |

1. Which age group(s) may be most affected by food-drug interactions (multiple answers can be selected):

- Pre-term
- Paediatrics
- Geriatrics
- Adults
- Teenagers
- Not sure

*Please select the most correct answer, to the best of your knowledge, without consulting additional resources. In other words, select the answer that is based on existing knowledge. If you are not sure of an answer, then please select NOT SURE rather than guessing.*

1. Which of the following foods lead to the treatment failure of the anticoagulant, warfarin (e.g. Cipla-Warfarin, Aspen Warfarin)?

| **Item** | **Yes** | **Not sure** | **No** |
| --- | --- | --- | --- |
| Beets |  |  |  |
| Garlic |  |  |  |
| Strawberry |  |  |  |
| Cranberry |  |  |  |
| Leafy greens |  |  |  |

1. Which of the following foods interact with tetracycline antibiotics (e.g. Tetralysal, Doxycyl), leading to treatment failure?

| **Item** | **Yes** | **Not sure** | **No** |
| --- | --- | --- | --- |
| Beans |  |  |  |
| Dairy products |  |  |  |
| Chicken |  |  |  |
| Nuts |  |  |  |
| Red meat |  |  |  |

1. Which of the following foods lead to hyperkalaemia when taken alongside angiotensin-converting enzyme inhibitors (e.g. enalapril (Vasotec®), lisinopril (Prinivil®, Zestril ®), benazepril (Lotensin®))?

| **Item** | **Yes** | **Not sure** | **No** |
| --- | --- | --- | --- |
| Pears |  |  |  |
| Bananas |  |  |  |
| Peaches |  |  |  |
| Oranges |  |  |  |
| Apples |  |  |  |

1. Which of the following foods decrease the therapeutic effects of the cardiovascular drug, digoxin (e.g. Lanoxin, Digox)?

| **Item** | **Yes** | **Not sure** | **No** |
| --- | --- | --- | --- |
| White rice |  |  |  |
| Avocados |  |  |  |
| Whole-grain bread |  |  |  |
| Celery |  |  |  |
| Watermelon |  |  |  |

1. Which of the following drugs' metabolism will be decreased by grapefruit juice?

| **Item** | **Yes** | **Not sure** | **No** |
| --- | --- | --- | --- |
| Simvastatin (e.g. Zocor; cholesterol-lowering statin) |  |  |  |
| Nifedipine (e.g. Adalat XL; calcium channel blocking antihypertensive) |  |  |  |
| Paracetamol (e.g. Panado; antipyretic) |  |  |  |
| Fexofenadine (Fexo; antihistamine) |  |  |  |
| Zanamivir (e.g. Tamiflu(oseltamivir); neuraminidase inhibitor antiviral) |  |  |  |

1. Which of the following foods may result in severe hypertension when administered with monoamine oxidase inhibitors (e.g. Depnil)?

| **Item** | **Yes** | **Not sure** | **No** |
| --- | --- | --- | --- |
| Pasta |  |  |  |
| Matured cheese |  |  |  |
| Sourdough bread |  |  |  |
| Processed meats |  |  |  |
| Cottage cheese |  |  |  |

1. Which of the following drugs will have a greater gastrotoxic effect if taken alongside alcohol?

| **Item** | **Yes** | **Not sure** | **No** |
| --- | --- | --- | --- |
| Antiretrovirals (e.g. Dolutegravir (e.g. Tivicay)) |  |  |  |
| Paracetamol (e.g. Panado |  |  |  |
| Antihistamines (e.g. Allergex, Texa Allergy, AP-Loratidine) |  |  |  |
| Warfarin (e.g. Sofarin) |  |  |  |
| Aspirin (e.g. Disprin) |  |  |  |

1. How should the following drugs be administered in terms of nutrition?

| **Item** | **Take on an empty stomach** | **With meals** | **Avoid iron** | **Avoid-high fat meals** | **Take with high-fat meals** | **Avoid calcium** | **Take at the same time every day** | **Not sure** |
| --- | --- | --- | --- | --- | --- | --- | --- | --- |
| Griseofulvin (e.g. Microcidal) |  |  |  |  |  |  |  |  |
| Efavirenz (e.g. Erige) |  |  |  |  |  |  |  |  |
| Dolutegravir (e.g. Tivicay) |  |  |  |  |  |  |  |  |

1. The following is true about the timing of these drugs relative to food:

| **Item** | **Before meals** | **With meals** | **After meals** | **Not sure** |
| --- | --- | --- | --- | --- |
| Ampicillin (e.g. Apen) |  |  |  |  |
| Glipizide (e.g. Minidiab) |  |  |  |  |
| Griseofulvin (e.g. Microcidal) |  |  |  |  |

1. How often do you encounter clinically-significant food-drug interactions in your practice? Please elaborate on why you think this is the case.

- Never; Why?
- Infrequently (once or twice a year); Why?
- Frequently (monthly); Why?
- Almost always (weekly); Why?
- Always (daily); Why?

1. Did you receive additional training on food-drug interactions? If yes, please describe the training.

- Yes
- No

1. Where do you source your information from when you require additional information on food-drug interactions? (multiple answers can be selected)

- Drug package inserts
- Textbooks
- Clinical websites
- Social media
- Mobile applications
- Other (specify)

1. Regarding package inserts used in consultations, please answer the following, and elaborate on your selection in the text box below.

| **Item** | **Always** | **Sometime** | **Never** | **Not applicable** |
| --- | --- | --- | --- | --- |
| Do you look at the medication list of patients during consultations? |  |  |  |  |
| Do you ask patients to bring their medication to consultations? |  |  |  |  |
| Do you use package inserts during consultations? |  |  |  |  |
| Do package inserts provide sufficient information? |  |  |  |  |

1. Regarding package inserts used in consultations, please answer the following, and elaborate on your selection in the text box below.

| **Item** | **Always** | **Sometime** | **Never** | **Not applicable** |
| --- | --- | --- | --- | --- |
| Do you look at the medication list of patients during consultations? |  |  |  |  |
| Do you ask patients to bring their medication to consultations? |  |  |  |  |
| Do you use package inserts during consultations? |  |  |  |  |
| Do package inserts provide sufficient information? |  |  |  |  |

1. Do you feel equipped to manage food-drug interactions? Please elaborate on your answer.

- Yes
- No

1. How do you feel your knowledge can be expanded on food-drug interactions? Please include any suggestions you may have and explain why they would work for you.
